# Conceptual understanding in science learning and the role of four psychometric variables: a person-centered approach

**DOI:** 10.3389/fpsyg.2023.1204868

**Published:** 2023-06-15

**Authors:** Julie Vaiopoulou, Themistocles Tsikalas, Dimitrios Stamovlasis, George Papageorgiou

**Affiliations:** ^1^School of Psychology, Aristotle University of Thessaloniki, Thessaloniki, Greece; ^2^Department of Education, University of Nicosia, Nicosia, Cyprus; ^3^Department of Primary Education, Democritus University of Thrace, Alexandroupolis, Greece; ^4^School of Philosophy and Education, Aristotle University of Thessaloniki, Thessaloniki, Greece

**Keywords:** conceptual change, divergent and convergent thinking, field-dependence/field-independence, logical thinking, latent class analysis, science learning, conceptual understanding, congitive variables

## Abstract

The present study investigated conceptual understanding in learning science in relation to four cognitive variables: logical thinking, field-dependence/field-independence, and divergent and convergent thinking. The participants were fifth- and sixth-grade elementary school pupils involved in different mental tasks, where they had to describe and interpret phenomena related to changes of matter. This brief report presents data from the students’ understanding of evaporation, and the method of analysis, a person-centered approach, is explicated. Latent class analysis (LCA) was applied to reveal distinct clusters of cases sharing similar patterns of responses. The use of LCA aligns with theoretical conjectures related to a stepwise conceptual change process, and the hypothetical steps correspond to the identified discrete latent classes (LCs). Subsequently, the LCs were associated with the four cognitive variables as covariates, thus providing empirical evidence for the role of the above-mentioned individual differences in children’s learning in sciences. Methodological issues and theoretical implications are discussed.

## 1. Introduction

The literature on children’s conceptual understanding of science is rich in reports focusing on erroneous or alternative ideas. Over decades, a crucial amount of knowledge has accrued, which has helped practitioners design teaching interventions, and academic scholars and theorists postulate assumptions about the nature of children’s representations. One of the most challenging themes in science teaching is the transformations of matter and related concepts (Liu and Lesniak, 2005, 2006; Papageorgiou and Johnson, 2005; García-Franco and Taber, 2009; Tsitsipis et al., 2010, 2012). Researchers’ strategies in probing children’s ideas include attempts to classify them and often identifying fundamental conceptions that facilitate or prevent the attainment of the scientific view. For example, the lack of understanding of the particulate nature of matter affects apprehending changes in the states of matter.

Interestingly, cross-cultural studies have revealed similar misconceptions, such as the content of the bubbles in boiling water being air, heat, carbon dioxide, or a mixture of hydrogen and oxygen, showing that children confuse such phenomena with chemical changes (Papageorgiou and Johnson, 2005; Talanquer, 2009; Papageorgiou et al., 2010). Inquiries regarding physical transformations of matter, such as evaporation, have shown that children’s cognitive obstacles originate from a lack of understanding of fundamental particle concepts and also from individual differences, e.g., logical thinking and cognitive styles (Johnson, 1998a,b; Tsitsipis et al., 2010, 2012). Researchers have also acknowledged that some kind of hierarchical levels of understanding exist in the progression from naïve ideas to the scientific view, often explicated via a specific level scheme (e.g., Hadenfeldt et al., 2014). Such putative levels imply a stepwise rather than a linear conceptual change process. Scholars from earlier times have postulated two theoretical perspectives about this. The first approach considers children’s knowledge before entirely acquiring the scientific view consistent or theory-like with explanatory power (Vosniadou and Brewer, 1992). The second approach supports the notion that these ideas are fragments of knowledge (p-prims) that come loosely together *in situ*, i.e., when a child needs to complete any task (DiSessa, 1993). The debate on these antithetical hypotheses is enduring since both have demonstrated empirical support. For the coherent knowledge hypothesis, explicit mental models have been proposed, while subsequent discussions have raised several methodological issues (Vosniadou and Brewer, 1992; Ioannides and Vosniadou, 2002; DiSessa et al., 2004; Nobes and Panagiotaki, 2007, 2009; Straatemeier et al., 2008). However, regardless of any argumentation, stepwise progression in learning processes remains an intriguing hypothesis, which, nevertheless, needs exploration via robust methodologies. One of the limitations of the past endeavors is that they relied on a simple analysis of empirical indicators, ignoring latent variable theory and contemporary models of psychological measurement (Bartholomew, 1987; Borsboom, 2008). Among them, latent class analysis (LCA) assumes that both observable and latent variables are categorical, and it is the proper measurement model for the coherent knowledge theory since the latter considers the children’s mental models as a categorical type of entities. Its applications have been demonstrated in exploratory and confirmatory approaches (Straatemeier et al., 2008; Vaiopoulou et al., 2017).

Moreover, most investigations on science learning have aimed merely to describe students’ hitches by labeling the difficulties of the specific topic without implementing independent variables within a more comprehensive explanatory framework. However, some researchers have found associations of conceptual change in science with several individual differences and notably cognitive variables, such as logical thinking, field dependence/independence, divergence, and convergence (BouJaoude et al., 2004; Tsitsipis et al., 2010, 2012; Tsikalas et al., 2014; Papageorgiou et al., 2016). In this endeavor, the selection of the above variables was made from among potential individual differences that were theory and evidence-based, while some others were excluded, e.g., working memory, which is associated with problem-solving and mathematical calculations.

The nature of children’s knowledge and the encountered cognitive obstacles, along with the role of individual differences in attaining conceptual change, are taken into account in designing pedagogical interventions, contributing to a theoretical framework aiming to identify, understand, and propose strategies to help children to overcome learning difficulties (Danili and Reid, 2004; Stamovlasis, 2006).

The present paper presents the implementation of the above cognitive variables within a person-centered approach and supports a stepwise conceptual change process, adding to the theoretical growth of the field. Next, a brief description of the above-mentioned psychometric variables follows.

### 1.1. Cognitive variables

#### 1.1.1. Logical thinking or formal reasoning

Logical thinking (LTH), or formal reasoning, is a Piagetian construct referring to one’s ability to successfully implement concrete and formal operational reasoning (Lawson, 1978, 1985, 1993). Measuring LTH includes proportional, combinational, and probabilistic thinking and reasoning related to the isolation and control of variables, conservation of mass, and displaced volume. Formal reasoning is associated with students’ achievement in numerous studies (Lawson, 1982; Niaz, 1996; Tsaparlis and Angelopoulos, 2000; Stamovlasis and Tsaparlis, 2001; BouJaoude et al., 2004; Tsitsipis et al., 2010, 2012) and, in general, it has been proven to be one of the most important predictors of academic performance.

#### 1.1.2. Field dependence/independence (FDI)

FDI is a cognitive style that concerns a person’s ability to separate relevant information from a complex and confusing context (Witkin et al., 1977). When individuals are dominated by a strong frame of reference and have difficulties separating an item from its context, they are characterized as field-dependent. On the contrary, individuals who can identify the item from the misleading context are called field-independent (Witkin and Goodenough, 1981). This cognitive style is considered continuous, and persons that fall in the middle of the FDI scale are defined as field-intermediate. FDI is a crucial cognitive variable involved in mental tasks, and learning science research has found that it is one of the main predictors of academic achievement (Tinajero and Páramo, 1998). In addition, FDI is a moderator variable within the information processing model due to the mental capacity and higher information processing ability required to process irrelevant information (Johnstone and Al-Naeme, 1991; Tsaparlis and Angelopoulos, 2000). Specifically, science education research has highlighted the critical role of field independence in conceptual understanding, learning, and problem-solving (Danili and Reid, 2004; Kang et al., 2005; Stamovlasis and Tsaparlis, 2005; Tsaparlis, 2005).

#### 1.1.3. Convergent and divergent thinking

Convergence (CONV) refers to the ability to focus on the one correct answer in order to solve a problem, whereas divergence (DIV) regards the ability to respond flexibly and successfully to problems requiring the production of multiple solutions (Child and Smithers, 1973). It is important to emphasize that even though the terms used indicate that they are opposites, they are two distinct cognitive styles (Heller, 2007) which represent two unique aspects of intelligence and can be complementary characteristics. Given that a scientific problem can be quite complex, both attributes are essential, i.e., divergent thinking in the first stages and convergence in the latter steps for decision-making and conclusions (Facaoaru, 1985; Heller, 2007). Indeed, in science education research, these psychometric variables are highly correlated to students’ achievement (Danili and Reid, 2006; Tsitsipis et al., 2010, 2012; Tsikalas et al., 2014).

## 2. Method

### 2.1. Theoretical and methodological issues

The present study fosters the theoretical perspective that advocates the existence of hierarchical progression steps toward conceptual change, i.e., the conceptual understanding process’s evolution is not linear but passes through discrete stages. This is a theoretical assumption that requires empirical support while it embraces the intriguing research question of whether a latent construct, such as conceptual understanding, is continuous or categorical (or taxon), regardless of the level of measurement of the observable indicators, which are the choice of the researcher. This question is the aim of *taxometrics*, a branch of psychometric modeling that seeks to identify if a latent construct is a *taxon*/categorical or dimensional/continuous variable (Haslam et al., 2012). Alternatively, the usual psychometric practice is the implementation of a suitable psychometric model, e.g., a factor model or a latent class model, and scrutinizing the model fit with empirical data. Therefore, the measurement model that ensures access to the latent variable in question should align with the theoretical conjectures. The appropriate method for investigating conceptual change under the theory of coherent mental models is LCA, a psychometric measurement model that considers both latent and observable variables at the nominal level of measurement. The above supports a cogent rationale for implementing LCA, especially when the empirical indices implemented are at the nominal level of measurement, identified as correct responses or misconceptions/incorrect responses. (Van der Maas and Straatemeier, 2008; Hofman et al., 2015). Furthermore, LCA is an adept procedure to identify distinct groups in the participants that provide distinct patterns of responses, which according to latent variable theory, are due to different latent representations. That is, the latent construct possessed by participants exhibits discrete levels rather than being a continuous variable. These represent stages of conceptual understanding that emerge from the analysis of the empirical data. LCA is an effective method to detect discrete mental representations, thinking patterns, and cognitive change (Van der Maas and Straatemeier, 2008; Hofman et al., 2015).

Moreover, exploring conceptual understanding requires an explanatory framework and independent predictors that could be statistically associated with the hypothetical stages. To this end, the present endeavor explores the theoretical conjecture of a stage-wise progression in conjunction with the explanatory role of four cognitive variables. The choice of the psychometric variables to be associated with the levels of understanding is theory-driven and evidence-based. These constructs operationalize mental resources theoretically involved in the cognitive processes of learning the subject matter. For instance, divergent thinking that is associated with imagination is required to comprehend phenomena of state change of matter at the sub-micro level of molecules. Analogous elaborations and association of all cognitive variables with the mental processes involved in learning sciences have been presented and can be found elsewhere, along with the empirical evidence (Tsitsipis et al., 2010, 2012).

There are two interrelated fundamental research hypotheses:

*H1.* The first relates to identifying the existence of steps or stages along the conceptual change progression in science learning.

*H2.* The second concerns the association of the four psychometric variables: logical thinking, field dependence/independence, divergent thinking, and convergent thinking, with children’s understanding of the changes of matter under study.

### 2.2. Participants and measures

Three hundred and seventy-five pupils taking a mandatory science course in the fifth and sixth grades of elementary school (aged 11–12, 50.9% boys and 49.1% girls) participated in the study. They were from different schools in the broad area of Macedonia, Greece. Data were collected during one school year through paper-pencil tests. Children were involved in various tasks related to matter and its transformational properties, and they had to describe and explain phenomena. The open-ended questionnaire included selected items utilized in related studies (Johnson, 1998a,b,c; Papageorgiou et al., 2010), preserving enhanced validity. The assessment instrument included illustrations that facilitated the representation of the phenomena in question. There were items emphasizing knowledge recall and the interpretation of phenomena, which comprised the dimensions of the underlying construct and were context specific depending on what had been taught. Three graders applied a marking scheme of incorrect (misconception), partially correct, and correct. This marking scheme provided three-level ordinal variables, treated as categorical and used as the input for the subsequent LCA. After discussing the ensuing discrepancies among the graders, the agreement was 100%.

In addition, the participants had to complete psychometric tests extensively used in science learning research over the last decades to measure the psychometric variables in question. Specifically:

Logical thinking (LTH) or formal reasoning was assessed using the Lawson test, measuring logical thinking levels (Lawson, 1978). A Cronbach’s alpha reliability coefficient of 0.78 was obtained.

Field dependence/independence (FDI) or disembedding ability is usually evaluated utilizing the Group Embedded Figures Test (GEFT); however, a similar test, the Hidden-Figures Test (HFT), was used, which has been devised and calibrated (Johnstone and El-Banna, 1986) from Witkin’s original test materials. Cronbach’s alpha reliability coefficient was 0.84.

Divergent thinking (DIV) was measured using a six-item test designed by Bahar (Bahar, 1999). Each item is substantially a mini-test in itself lasting for 2–5 min that asks students to: generate words with similar meanings to those given (Test 1); construct up to four sentences using the words in the form as given (Test 2); draw up to five different sketches relevant to a given idea (Test 3); write down as many things as possible that have a common trait (Test 4); write down as many words as possible that begin with one specific letter and end with another specific letter (Test 5); and list all the ideas about a given topic (test 6). This instrument was used first with Greek students by Danili and Reid (2006) and by Tsitsipis et al. (2010). Cronbach’s alpha reliability coefficient was 0.74.

Convergent thinking (CONV) was assessed using a five-item timed test (Hindal et al., 2009) translated into Greek and modified to fit Greek idioms. Students answered each question separately for a total time of 20 min. Test 1 asked students to find two patterns that link to a group of words given (question 1), to form two words from the letters given (question 2), and to write down and explain a number missing from three sequences given (question 3). For Test 2, students had to read about a topic and classify three main ideas in a diagram. The task of Test 3 was to pick out a different object from a group of four and explain why they selected it. Test 4 comprised writing down two true things for all four given graphs. For Test 5, students marked a route on a map and described it in a few words. For the present sample, Cronbach’s alpha reliability coefficient was 0.77.

### 2.3. Latent class analysis

LCA is a psychometric method that uses Bayesian statistics to classify a set of responses or observable categories into clusters or latent classes (LCs) based on a set of conditional probabilities, which are the probabilities of having a response pattern y given the specific latent class c (Clogg, 1995; Dayton, 1998). Based on several criteria, such as the classification error, entropy *R*^2^, the number of parameters, likelihood ratio statistic (L2), Bayesian information criterion (BIC), Akaike’s information criterion (AIC), degrees of freedom, and bootstrapped *p*-value, the researcher decides on LC model fitting to empirical data (Magidson and Vermunt, 2004).

An advantage of LCA is the option to include external variables in the model, functioning as covariates or distal outcomes. Incorporating such variables defines an LC model comprising two parts: the measurement part, consisting of information on how the clusters are derived, and the structural part, dealing with the relationships between the ensued clusters and the external variables (Bakk et al., 2013). The process to carry out LCA can be either one-step or stepwise. The latter method includes three steps: (i) The identification of the underlying latent variable based on a set of indicators, (ii) The assignment of cases (individuals) to latent classes, and (iii) The analysis of the results into class membership and the covariates, accordingly. The stepwise method is preferable when the predictive validity of the covariate is of interest, while different classification methods can be applied along with bias-correction procedures (Bolck et al., 2004; Vermunt, 2010).

LCA has numerous applications in a wide range of disciplines and fields. For example, in educational research, LCA has been proven effective in deriving participants’ profiles and answering challenging research questions. Some indicative investigations include exploring students’ predictions and explanations of physical phenomena (Schneider and Hardy, 2013; van Schaik et al., 2020) and testing contradictory theoretical perspectives, such as the coherence vs. fragmented knowledge hypotheses (Straatemeier et al., 2008; Vaiopoulou and Papageorgiou, 2018; Stamovlasis et al., 2020).

## 3. Results

### 3.1. The ensuing latent classes

This short report analyzes and presents part of the data, specifically children’s responses to tasks related to the evaporation phenomenon, via LCA. The analysis, including the five corresponding items, led to the results in Table 1, where the three latent classes (LC) were identified as the best parsimonious model (entropy-*R^2^* = 0.87, df = 324, classification error = 0.0445, BIC = 2343.12, Npar = 40).

**Table 1:** Evaporation—latent class analysis (LCA).

	LL	BIC(LL)	Npar	*L* ^2^	df	*p*-value	Class. err.	Entropy *R*^2^
1-Cluster	−1225.1	2509.16	10	2450.19	354	1.0	0	1
2-Cluster	−1111.18	2369.79	25	2222.36	339	0.31	0.0555	0.80
3-Cluster	−1053.62	2343.12	40	2107.23	324	0.35	0.0445	0.87
4-Cluster	−1,021	2366.35	55	2042.01	309	0.21	0.0653	0.84
5-Cluster	−1006.32	2425.45	70	2012.65	294	0.19	0.1081	0.80

A set of conditional probabilities concerning the responses in each item characterizes each of the ensuing latent classes (LC). In a graph showing the cumulative probabilities of a latent class, it is possible to visualize a pattern depicting the consistency of responses regarding the levels of judgment: correct, partially correct, and incorrect. These patterns designate the progression level; that is, a pattern where the incorrect responses (misconceptions) prevail denotes a lower level than a pattern dominated by correct responses.

**Figure 1:**
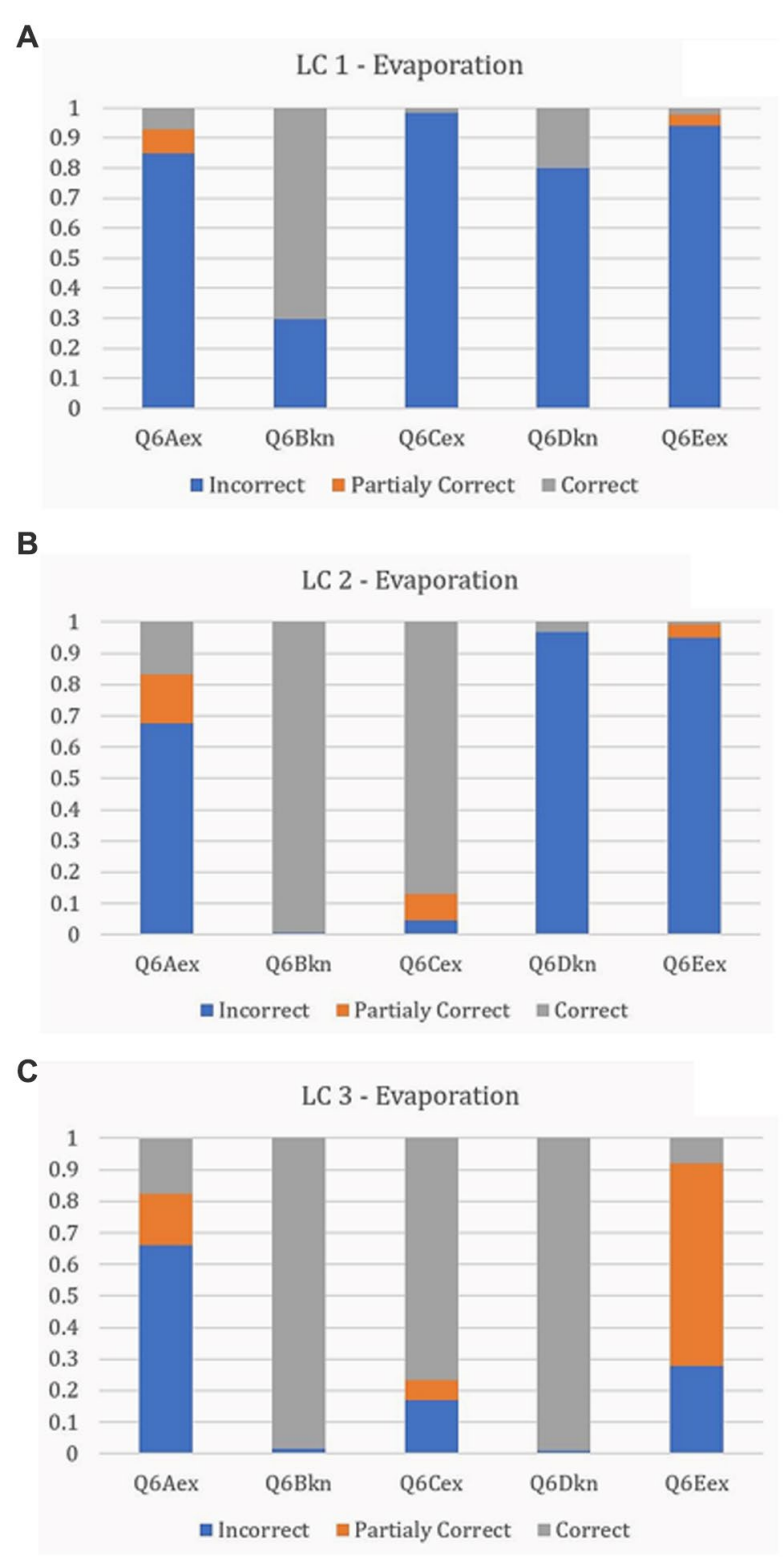
The ensuing latent classes.

Figure 1 depicts the three latent classes. LC1 (20.54%) includes children who provided primarily incorrect responses, i.e., misconceptions (Figure 1A). LC2 (43.04%) consists of children who, compared to LC1, provided some correct or partially correct answers and demonstrated a nuanced understanding. LC2 possesses an evident degree of heterogeneity regarding the responses in the five items, given that the analysis provided a pattern with a mixture of incorrect, correct, and partially correct answers (Figure 1B). LC3 (36.42%) includes children mainly providing correct responses. This pattern, compared to LC2, appears less fragmented and relatively homogeneous, with the correct answers prevailing (Figure 1C).

The three LCs are evidently hierarchical, representing three stages that children could have reached through a learning process, and these groups are the categories of the latent variable under investigation. The crucial question is how they are associated with the cognitive variables, which are anticipated to function as predictors of learning outcomes.

### 3.2. Effect of the four cognitive variables on latent classes

In the next step, latent classes were associated with the four covariates, considered independent variables, and predicted the LC membership.

Table 2 shows the effects of cognitive variables on the three latent classes. LC1 is negatively associated with LTH (*b* = −0.742, *p* < 0.0001), FDI (*b* = −0.183, *p* < 0.05), DIV (*b* = −0.457, *p* < 0.001), and CONV (*b* = −0.675, *p* < 0.001). On the other hand, LC3 is positively associated with all covariates: LTH (*b* = 0.555, *p* < 0.0001), FDI (*b* = 0.217, *p* < 0.01), DIV (*b* = 0.433, *p* < 0.001), and CONV (*b* = 0.559, *p* < 0.001). In between LC1 and LC3, LC2 is non-significantly associated with the psychometric variables with the exception of LTH (*b* = 0.187, *p* < 0.01).

**Table 2:** Effects of covariates on the latent classes.

		Latent class 1	Latent class 2	Latent class 3
Covariates	LTH	−0.742***	0.187**	0.555***
	FDI	−0.183*	−0.034	0.217**
	DIV	−0.457***	0.023	0.433***
	CONV	−0.675***	0.116	0.559***

*p < 0.05, **p < 0.01, ***p < 0.001.

In conclusion, LCA applied to children’s responses revealed distinct clusters corresponding to discrete conceptual understanding levels. The roles of the four cognitive variables examined as covariates of LC memberships follow a consistent pattern, associated negatively and positively with the lower and higher levels of conceptual understanding, respectively. The interpretation of the coefficient in Table 2 is analogous to the logistic regression coefficients, i.e., the higher the LTH value, the higher the odds of belonging to LC3. The above relationships indicate the crucial role of mental resources in the underlying learning process and imply their involvement in forming various performance levels.

## 4. Discussion

### 4.1. Interpretation of the findings and theoretical perspectives

LCA suggests that the latent variable under study can be considered categorical. In this study, it revealed certain clusters/LC where, in each LC, children shared similar response patterns, which according to psychometric measurement theory are due to a latent construct that is the common cause of the observed responses. Thus, children within the same LC possess the same latent mental representations. LCA was used in an exploratory mode since the clusters were not defined *a priori*, but they emerged from the analysis. Note that elsewhere (Straatemeier et al., 2008; Vaiopoulou et al., 2017; Vaiopoulou and Papageorgiou, 2018; Stamovlasis et al., 2020), the method was used in a confirmatory mode to validate specific mental models explicitly stated in the literature (Vosniadou and Brewer, 1992; Ioannides and Vosniadou, 2002). These proposed mental models/latent classes were identified and characterized by specific concurrent misconceptions, which, in fact, have been used in the classification process to obtain those mental models. To construct the hypothesized mental models via exploratory processes while proposing them as the stages being sought. This approach adheres to the epistemological view of *substance* philosophy, contrary to the *process* philosophy (Seibt, 2022) which is the view fostered in the present interpretation.

Given the existence of discrete levels of understanding and their association with cognitive variables, it is essential to pursue an interpretation of the findings and explicate the nature of these stages in conjunction with their predictors. The insight here is to reconsider the involvement of cognitive variables and explain the findings by fostering a process-based approach that identifies cognitive phenomena situated and time-dependent in nature (Van Geert and de Ruiter, 2022). The meta-theoretical framework of complex adaptive systems (CDS) and non-linear dynamics may provide a suitable vocabulary description of the process under investigation. The well-known concept of *attractor* fits the notion of the stages sought in the present endeavor. An attractor is a stable state or a set of stages toward which a system’s evolution tends to move (Freeman and Barrie, 1994; Kelso, 1995), while the changes in the system are understood as transitions between attractors. Ergo, the CDS framework can reasonably provide a theoretical justification for the existence of discrete stages.

If the detected LCs are viewed or related to a kind of *attractor state*, their association with psychometric variables implies that the corresponding mental resources are part of or are involved in forming the attractor. From the CDS perspective, the mental operators corresponding to LTH, FDI, DIV, and CONV act successively in a dynamical and iterative process, where potential failed synergies among them trap the mind into a lower performance attractor stage. Dynamical causes stemming from insufficient cooperation among mental resources might lead to a sub-optimal conceptual understanding, a state that could be characterized as a conceptual attractor. Such a description explicitly assumes that the temporal outcome is not an additive function of the contributing components, i.e., the underlying process is not linear.

The above explanation from the CDS perspective adds to conceptual change research by proposing the operation of mental resources and their dynamical co-actions as contributing to the formation of dynamical attractors, in which the LCs are detected as distinct stages.

### 4.2. Limitations and future research

The present research has limitations that originate from the cross-sectional type of design and the opportunity sampling procedure. Given that it is the first endeavor investigating participants of the young age of 11–12, the findings need replication with different samples. Moreover, the theoretical interpretation attempted via CDS needs further refinement and a complete elaboration to establish a definite amalgamation of conceptual change theories within the complexity theory and non-linear dynamical system framework. Additionally, this perspective’s development needs empirical evidence by means of non-linear methodological analyses. Research has shown that the psychometric constructs under investigation that operationalize active mental resources are predictive variables in children’s cognitive performance, while applications of catastrophe theory models have associated them with non-linear changes that explicate transitions between stages (Stamovlasis, 2006, 2011; Stamovlasis and Tsaparlis, 2012; Vaiopoulou et al., 2021; Stamovlasis et al., 2023). Thus, a following inquiry may seek potential non-linear changes between the discovered stages (or attractors)—that is, transitions, for instance, between LC1 and LC2—revealed in the various cognitive tasks as a function of the cognitive variables under study.

## Data availability statement

The raw data supporting the conclusions of this article will be made available by the authors, without undue reservation.

## Ethics statement

The studies involving human participants were reviewed and approved by the Greek Ministry of Education (protocol code “υπ. Αριθ. Φ. 15/545/83146/Γ1/19-7-12” on 19 July 2012). Written informed consent to participate in this study was provided by the participants’ legal guardian/next of kin.

## Author contributions

JV, TT, DS, and GP contributed to the conception and design of the study and wrote sections of the manuscript. JV organized the database, performed the statistical analysis, and wrote the first draft of the manuscript. All authors contributed to the article and approved the submitted version.

## Conflict of interest

The authors declare that the research was conducted in the absence of any commercial or financial relationships that could be construed as a potential conflict of interest.

## Publisher’s note

All claims expressed in this article are solely those of the authors and do not necessarily represent those of their affiliated organizations, or those of the publisher, the editors and the reviewers. Any product that may be evaluated in this article, or claim that may be made by its manufacturer, is not guaranteed or endorsed by the publisher.
